# Continuous Classification of Locomotion in Response to Task Complexity and Anticipatory State

**DOI:** 10.3389/fbioe.2021.628050

**Published:** 2021-04-22

**Authors:** Mahdieh Kazemimoghadam, Nicholas P. Fey

**Affiliations:** ^1^Department of Bioengineering, The University of Texas at Dallas, Richardson, TX, United States; ^2^Walker Department of Mechanical Engineering, The University of Texas at Austin, Austin, TX, United States

**Keywords:** task anticipation, changes of direction, intent recognition, training paradigm, analysis window size

## Abstract

**Objective:**

Intent recognition in lower-extremity assistive devices (e.g., prostheses and exoskeletons) is typically limited to either recognition of steady-state locomotion or changes of terrain (e.g., level ground to stair) occurring in a straight-line path and under anticipated condition. Stability is highly affected during non-steady changes of direction such as cuts especially when they are unanticipated, posing high risk of fall-related injuries. Here, we studied the influence of changes of direction and user anticipation on task recognition, and accordingly introduced classification schemes accommodating such effects.

**Methods:**

A linear discriminant analysis (LDA) classifier continuously classified straight-line walking, sidestep/crossover cuts (single transitions), and cuts-to-stair locomotion (mixed transitions) performed under varied task anticipatory conditions. Training paradigms with varying levels of anticipated/unanticipated exposures and analysis windows of size 100–600 ms were examined.

**Results:**

More accurate classification of anticipated relative to unanticipated tasks was observed. Including bouts of target task in the training data was necessary to improve generalization to unanticipated locomotion. Only up to two bouts of target task were sufficient to reduce errors to <20% in unanticipated mixed transitions, whereas, in single transitions and straight walking, substantial unanticipated information (i.e., five bouts) was necessary to achieve similar outcomes. Window size modifications did not have a significant influence on classification performance.

**Conclusion:**

Adjusting the training paradigm helps to achieve classification schemes capable of adapting to changes of direction and task anticipatory state.

**Significance:**

The findings could provide insight into developing classification schemes that can adapt to changes of direction and user anticipation. They could inform intent recognition strategies for controlling lower-limb assistive to robustly handle “unknown” circumstances, and thus deliver increased level of reliability and safety.

## Introduction

Locomotion is initiated by robust motor patterns within the nervous system and involves synchronized activity of muscles and the skeletal system triggering a set of cyclic movements (Chiel and Beer, 1997). Neuromuscular diseases, aging population, and limb amputation due to trauma or disease are the factors that can cause physical limitations and lower limb dysfunction (McDonald, 2002; Cesari et al., 2005; Ziegler-Graham et al., 2008). Advanced lower-limb assistive devices (i.e., prostheses and exoskeletons) are being developed to better aid individuals with motor impairments/amputation in performing daily activities. For instance, microcontroller-based/powered assistive devices have the potential to provide intuitive transitions between locomotor tasks and allow automatic and smooth “steering” (Huang et al., 2011; Young and Ferris, 2017). In order to achieve seamless integration of the device with the wearer, gait information needs to be acquired real time, and locomotor mode should be instantaneously identified by the control algorithms (Varol et al., 2010; Jiménez-Fabián and Verlinden, 2012). Intent recognition using machine learning has been the popular methodology to infer user intention for the device, and to effectively identify target locomotion modes (Young et al., 2013; Hargrove et al., 2015).

However, to date, intent recognition strategies have been primarily limited to predicting user movements during straight walking including steady state locomotion or transitions from one terrain to another, e.g., level ground to ramp or stairs locomotion (Au et al., 2008; Tkach and Hargrove, 2013; Chen et al., 2014). Control approaches become more sophisticated due to the fact that not only changes of terrain, but also changes of direction and non-steady maneuvers such as turns/cuts are among prevalent locomotor tasks performed in daily living and many sport/recreation activities. Glaister et al. (2007) conducted a study to investigate how often non-straight locomotion is performed in daily living. They mimicked the activities performed most often in a typical day and demonstrated that turning constitute approximately 50% of all steps taken. Depending on the frequency of performing certain tasks, the percentage of such non-straight steps could be even larger. For instance, performing tasks with tighter constraints or high demand require subjects to change direction/turn more frequently (Sedgman et al., 1994). Also, studies showed that changes of direction and steering body in a new direction was the dominant choice among participants for avoiding obstacles (Jansen et al., 2011). The level and rate of human adaptation to the use of assistive devices such as lower limb prosthesis and exoskeletons are strongly related to the ability of the device to provide natural, safe, and stable response for a wide range of daily living activities and environments. Quick and correct identification of demanding transitions such as changes of direction would benefit microcontroller-based lower limb prosthesis as well as lower limb exoskeletons designed to help people with walking impairments (Farris et al., 2014) and to enhance the physical abilities of healthy subjects (Zoss et al., 2006). Accurate detection of such tasks would allow an assistive device to implement a proper mechanical response. The study could benefit the control/design of new prosthetic/orthotic device solutions for use during demanding and destabilizing locomotor tasks that continue to challenge currently available assistive technologies for the lower limb.

The most frequent turn styles include non-steady sidestep and crossover cuts. During crossover cut, the swing (trailing) leg crosses over the stance leg, toward the new direction while in sidestep cut, the swing leg is placed laterally, away from the stance leg. These tasks demand high level of coordination and modification of individual’s kinematics to change the plane of progression from straight to the intended direction of movement, and induce high levels of braking forces and muscle activation (Houck, 2003; Potter et al., 2014). It has been reported that stability is significantly affected during changes of direction, posing higher risks of injury compared to straight walking, especially when they are performed in *unanticipated* manner (Weinhandl et al., 2013; Wu et al., 2015). When a maneuver is self-initiated, users make preparatory locomotor adaptations to kinematics such as frontal plane hip and knee moments, as well as step size prior to the transition, to optimize their gait pattern and increase their margin of stability (Rand and Ohtsuki, 2000; Hak et al., 2013). In contrast, in unanticipated conditions, such as when an auditory or visual stimulus occurs, the level of preparatory adaptations decreases, which could inevitably increase the risk of stumbles and falls (Rand and Ohtsuki, 2000; Houck, 2003; Iguchi et al., 2014; Stokes et al., 2017). Nevertheless, current locomotion recognition approaches implemented in lower-limb assistive devices assume that the tasks are preplanned and the user can *anticipate* the events, which does not accurately represent real environments users experience.

Current assumption in controlling assistive devices considering identical input signals for anticipated and unanticipated locomotor tasks may eventually constrain developing proper task identification strategies to prevent falls. Furthermore, the fact that most patient populations suffering from motor impairment are also diagnosed with sensory or cognitive deficits (Dietz, 2002) make the previously mentioned assumptions further questionable. Increased task complexity is another factor placing high demands on the neuromuscular control system. For example, a sudden change of direction (cutting) or turning transition requires the forward linear momentum of the body’s center-of-mass to be changed by a horizontal angle of adjustment (Cao et al., 1998); but a more complex, combined cut and stair-ascent transition requires both horizontal and vertical alterations. Thus, new approaches are required to predict user intents during locomotion when they encounter unanticipated variations in the terrain and need to perform complex and non-steady transitions.

Sliding-window based segmentation has been utilized in computerized assistive devices to divide neuromechanical signals, and to identify the locomotion mode associated with each small snapshot of the gait cycle (Young et al., 2014a,b). Nonetheless, there has not been much information on comparing varying analysis window lengths during more complex locomotion such as unanticipated cutting tasks where quick biomechanical adjustments are induced. Linear discriminant analysis (LDA) has been the benchmark for locomotion mode recognition in robotic lower-limb assistive devices (Young et al., 2014a,b; Hargrove et al., 2015), and has demonstrated a good compromise between computational efficiency and recognition accuracy. The simplicity of the model in both preparation and application allows easy adaptation of the algorithm to new problems in real-time implementation. In any machine learning technique, appropriate selection of training data plays a significant role in achieving better classification outcome and saves computational efforts, whereas, poor training data allocation results in classifiers that do not generalize well to target samples (Bishop, 2006). This arises the question that how anticipatory state of the classifier’s training data would impact its generalization to unanticipated target tasks.

In this study, an LDA classifier was used to continuously predict straight-line walking, sidestep and crossover cuts, and cuts-to-stair transitions performed under altered task anticipatory states. We conducted an offline analysis using accelerographic, gyroscopic, and joint angle signals from the lower-limb and torso, recorded from able-bodied individuals as they performed the tasks. We expect that these variables can be provided with relative ease in an implementation of a lower-limb assistive technologies. Many signals such as this are already provided by embedded sensors on many assistive platforms. For instance, in the Vanderbilt powered knee-ankle prosthesis, a six-axis IMU captured linear acceleration and angular velocity of the shank. Position and velocity signals of knee and ankle were also incorporated for intent recognition (Young et al., 2014b). In exoskeletons, depending on the intended application input data configuration varies. For instance, Hybrid Assistive Limb uses hip and knee joint angles as the input signals for the controller (Kawamoto and Sankai, 2005), while H2 robotic exoskeleton functions utilizing angular position data from right and left hip, knee, and ankle (Bortole et al., 2015). In addition, other signals that we compared in our study can be readily provided by wearable IMU sensors or post-processing of these raw signals, which are routinely implemented for real-time control (Lenzi et al., 2018; Duraffourg et al., 2019).

Our primary objective was to investigate the effects of task anticipatory state (i.e., whether or not a transition was anticipated) on recognition accuracy. For this purpose, the classifier was trained with anticipated locomotor modes, and recognition accuracy of anticipated and unanticipated tasks were compared. Subsequently, varying repetitions (bouts) of target unanticipated locomotion were included in the training data to evaluate its influence on the generalization of the classifier to a different anticipatory state. Further, a set of sliding and overlapping windows of size 100–600 ms with a 25 ms increment were tested within each training paradigm. We hypothesized that anticipatory state of the locomotion mode would impact recognition accuracy. We further hypothesized that using larger analysis window sizes and including bouts of target task to the training data, would lead to improved recognition of unanticipated locomotor modes.

## Materials and Methods

### Subjects and Data Collection

Data were collected from five healthy subjects (four females, one male, average age: 27.7 ± 3.8 years, mass: 52.6 ± 2.8 kg, height: 1.68 ± 0.06 m), with no history of neuromuscular impairments or injuries. The experimental procedures used for this study were reviewed and approved by the Institutional Review Board (STU00060101), and subjects provided written consent before participating in the experiments.

**FIGURE 1:**
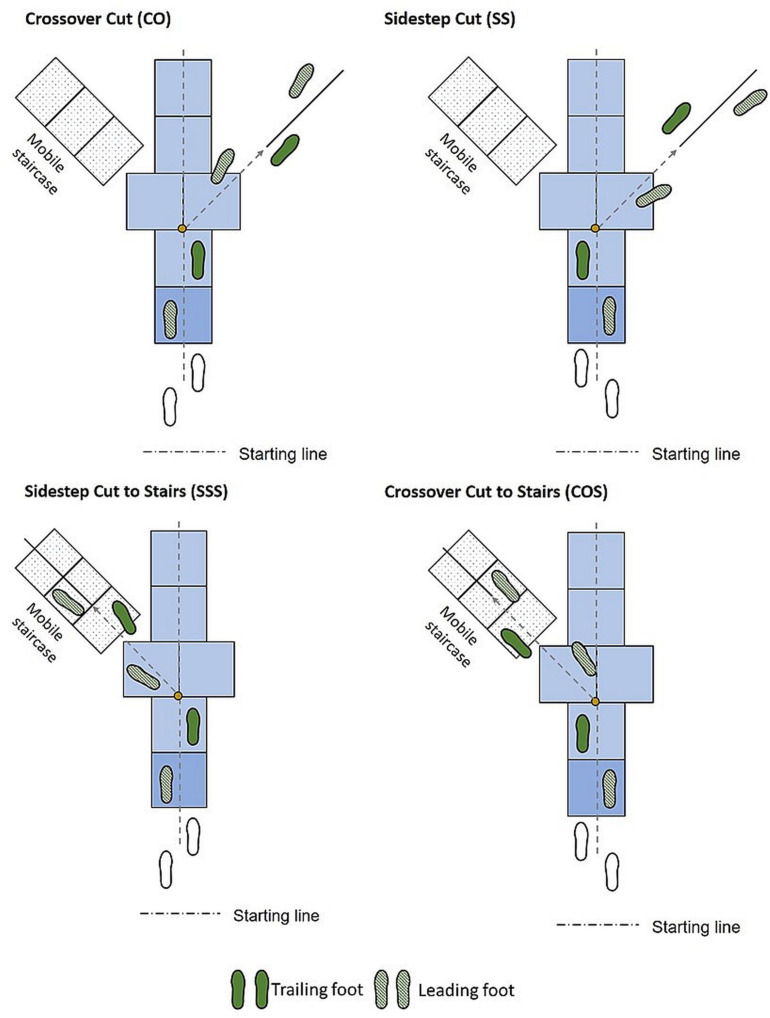
Experimental setup for locomotor transitions. During crossover cut (CO), the swing (trailing) leg crosses over the stance leg, toward the new direction while in sidestep cut (SS), the swing leg is placed laterally, away from the stance leg. In crossover to stair ascent (COS) and sidestep to stair ascent (SSS), following cuts subjects transition to staircase at 45° to the left.

**FIGURE 2:**
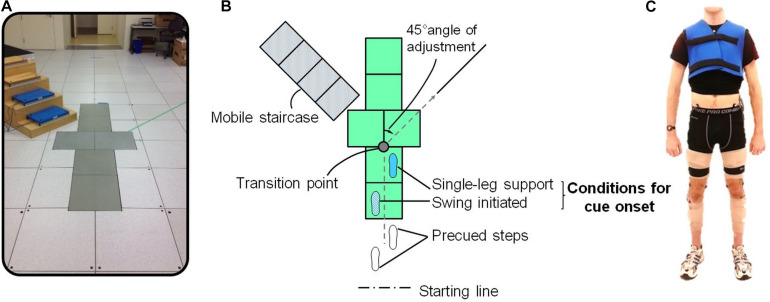
Experimental setup for locomotor tasks. **(A)** Lab setup for level-ground walking, sidestep and crossover cuts, and cut-to-stair-ascent locomotion. A custom built 4-step staircase was used for the tasks with the stair conditions with risers and treads of dimension 7.5 in and 12 in, respectively. For ascent and descent conditions, the length of the walkway preceding the staircase was 6.2 m and 5.2 m, respectively. **(B)** Subjects enter a locomotor transition at single leg support. The transition point and path for cut transitions were shown on the walkway. **(C)** Forty-two reflective markers including four clusters of markers connected via thermoplastic shells were placed on trunk, pelvis, and legs. Kinematic marker data were measured at 120 Hz using a 10-camera motion capture system (Motion Lab Systems, Inc.). Cameras were placed circumferentially along the wall of the motion capture lab, which was roughly 1,000 ft^2^ in size.

Subjects were instructed to perform five different locomotor transitions. They were asked to walk straight over the ground at comfortable speed (W), perform crossover (CO) and sidestep (SS) cuts, as well as crossover to stair-ascent (COS) and sidestep to stair-ascent (SSS) (Figure 1). All the locomotor modes were performed under both anticipated (A) and unanticipated (UA) conditions. Changes of transition style was defined as CO versus SS, and changes of task complexity was defined as cut versus cut to stair ascent. The lab setup was comprised of a level straight walkway, a level 45° walkway to the right for cutting, and a portable staircase at 45° to the left for the maneuvers with stair transitions (Figures 2A,B). A custom built 4-step staircase was used for the tasks with the stair conditions with risers and treads of dimension 7.5 and 12 in., respectively. For ascent and descent conditions, the length of the walkway preceding the staircase was 6.2 and 5.2 m, respectively (Peng et al., 2016).

The tasks with and without stair ascent were introduced as “mixed” and “single” transitions, respectively. Each subject completed straight-walking trials, followed by five trials for each anticipated cut style and complexity. Following that, unanticipated trials were captured when a randomized auditory cue for “walk,” “cut,” and “stair” was given at the beginning of the trailing leg swing phase and half a step before the marked transition point on the terrain (Figure 2B). Similar to anticipated trials, unanticipated data was comprised of unanticipated trails of level walking and each task complexity and style. The position of each leg when the cue was given was used to define the trailing and leading legs. Each trial was initiated by the first trailing leg heel strike (THS1). To execute cut, subjects started the transition at the first trailing leg toe off (TTO1) and ended with the second trailing leg heel strike (THS2). In mixed transitions, stair ascent was initiated by the second leading leg toe off (LTO2) and ended with the third trailing leg heel strike (THS3). Forty-two reflective markers including four clusters of markers connected via thermoplastic shells were placed on trunk, pelvis, and legs (Figure 2C). Kinematic marker data were measured at 120 Hz using a 10-camera motion capture system (Motion Lab Systems, Inc.). Cameras were placed circumferentially along the wall of the motion capture lab, which was roughly 1,000 ft^2^ in size.

### Signal Processing and Training Paradigms

Kinematic motion data were collected at 120 Hz from the lower-limb and trunk (Motion Lab Systems, Inc.), and corresponding signals were computed including linear acceleration and angular velocity from lower-limb segments (i.e., bilateral foot, shank, thigh), trunk, and pelvis as well as ankle dorsi-plantarflexion angle, knee flexion-extension angle, and hip rotation angles in three dimensions using Visual3D (C-Motion, Germantown, MD, United States). Segment linear acceleration and angular velocity were expressed in each segment’s reference frame, and joint angles were computed/expressed in each of the proximal segment’s reference frame. Data were exported to MATLAB (Mathworks, Natick, MA, United States) for further analysis. Each kinematic signal was divided into sliding and overlapping analysis windows. Six time-domain features including minimum, maximum, mean, standard deviation, first, and last sample of each window were extracted. These features are computationally inexpensive and have functioned relatively well in real-time control of lower-limb assistive device (Varol et al., 2010; Huang et al., 2011; Young et al., 2014a,b). A linear discriminant analysis (LDA) classifier was used to continuously classify the locomotion modes by associating each analysis window to its predicted mode.

In order to predict anticipated tasks, the nominal windows of size 300 ms with 25 ms increment was selected, and LDA was trained on anticipated trials of straight walking, cuts, and cuts-to-stair locomotion. Prediction of unanticipated locomotion modes was attempted using five different training paradigms with varying exposures of anticipated and unanticipated data as follows.

• Zero-Trial: Only anticipated training data.
• One-Trial: Anticipated training data plus one bout of unanticipated target task.
• Two-Trials: Anticipated training data plus two bouts of unanticipated target task.
• All-UA: Only unanticipated training data.
• UA-A: Anticipated and unanticipated training data.

To investigate the influence of analysis window size on recognition of unanticipated locomotion modes, windows of size 100–600 ms with 25 ms increment were tested within each training paradigm. The range of overlapping windows was selected according to the most common window sizes used in previous relevant studies (Young et al., 2014a,b).

### System Evaluation

All trials were synchronized at the first trailing leg toe off (TTO1) where a locomotor transition was initiated. For each task, system evaluation was performed using leave-one-out cross validation on each subject’s data, and the results were then averaged across the subjects. Classification accuracy was defined as the number of correctly identified windows divided by the total number of windows starting TTO1 to the end of the locomotion, reported in percentage (1).

(1) $$\begin{array}{c} \text{Accuracy}=\\ \frac{\text{number}\,\text{of}\,\text{correctly}\,\text{classified}\,\text{windows}\,\text{begining}\,\text{toe}\,\text{off}}{\text{total}\,\text{number}\,\text{of}\,\text{windows}}\times 100 \end{array}$$

Classification error patterns over time were obtained by allocating either 0 or 100 to every single analysis window. A window was marked as 0 when the predicted class was the same as the target class and was considered 100 when it was different. Corresponding windows were then averaged and error trends versus time were plotted. Overall classification accuracy and error rates were calculated across all the tasks. Confusion matrix (CM) was also computed to describe the error distribution among the locomotion modes due to misclassification (2).

(2) $$\text{CM}=\left[\begin{array}{ccccc} a_{11} & a_{12} & \cdots & \cdots & a_{15}\\ \cdots & a_{22} & \cdots & \cdots & a_{25}\\ \cdots & \cdots & a_{33} & \cdots & \cdots \\ \cdots & a_{42} & \cdots & a_{44} & \cdots \\ a_{51} & \cdots & \cdots & \cdots & a_{55} \end{array}\right]$$

Each element of matrix CM was defined as (3).

(3) $$a_{i\,j}=\frac{\text{number}\,\text{ofwindows}\,\text{in}\,\text{class}\,i\,\text{classified}\,\text{as}\,\text{class}\,j}{\text{total}\,\text{number}\,\text{of}\,\text{windows}\,\text{in}\,\text{class}\,i}\times 100\;$$

Where the diagonal elements in confusion matrix represent the classification accuracy for the target locomotion, and off-diagonal elements represent the levels of misclassification.

The Shapiro-Wilk test was used to check the normality assumption of the data. Then, two-way ANOVA was performed with the accuracy rate as the response variable, and training paradigm and analysis window length as fixed factors. If the ANOVA revealed a significant result, *post hoc* testing using pairwise comparison with Bonferroni correction was performed. The α level was set to be 0.01 for all tests. Power analysis was also performed for statistically significant results. At an α of 0.01, the null hypothesis (i.e., there is no true difference) was rejected when statistical power exceeded 80%.

## Results

### Recognition of Anticipated Locomotor Tasks

**FIGURE 3:**
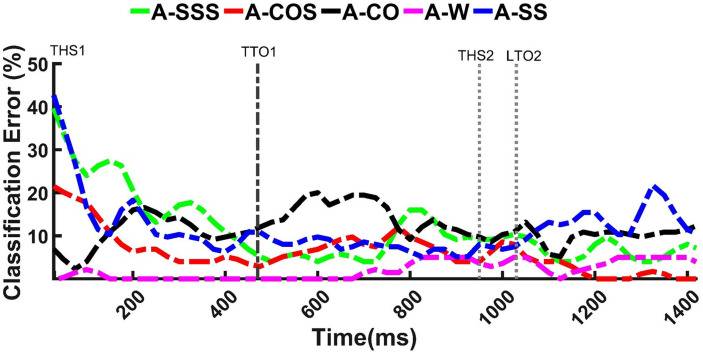
Classification error versus time for anticipated straight walking (A-W), cuts (A-CO, A-SS), and cuts to stair ascent (A-COS, A-SSS). The graphs demonstrate the possibility of predicting the tasks with error rates of <20% as early as 200 ms prior to entering the transition (TTO1). Each trial was initiated by the first trailing leg heel strike (THS1). To execute cut, subjects started the transition at the first trailing leg toe off (TTO1) and ended with the second trailing leg heel strike (THS2). LTO2 stands for the second leading leg toe off. Gait events are the average across subjects. *X*-axis data was shown up to the length of the shortest trial.

Classification of anticipated tasks were attempted using Zero-Trial training paradigm and nominal window size of 300 ms with 25 ms increment. Higher levels of accuracy were reported for anticipated mixed transitions (A-COS, A-SSS) and straight walking compared to single transitions (A-CO, A-SS) which was mainly due to misclassification of A-CO and A-SS as each other (∼13%) (Table 1). Prediction of anticipated tasks with error rates of ≤20% appeared to be possible as early as 200 ms prior to entering the locomotor transition (i.e., trailing leg toe off, TTO1) (Figure 3). A-SS and A-SSS error rates decreased from 35% at the beginning of the trial to <20% at approximately 200 ms prior to TTO1 and remained almost steady towards the end of the trial. A-COS classification error showed a downward trend over time, with error reaching ∼5% at 200 ms prior to TTO1. A-CO error appeared to remain below 20% during task progression. A-W was predicted best, with errors ranging 0–5% over time.

**TABLE 1:** Confusion matrix for anticipated locomotor tasks.

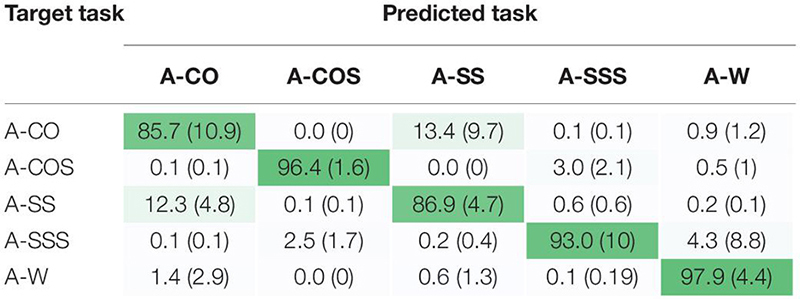

*Values represent mean (standard deviation). Diagonal darker elements show classification accuracies for the target locomotion, and off-diagonal elements are indicative of misclassification.*

### Recognition of Unanticipated Locomotor Tasks Using Varying Analysis Window Sizes and Training Paradigms

**FIGURE 4:**
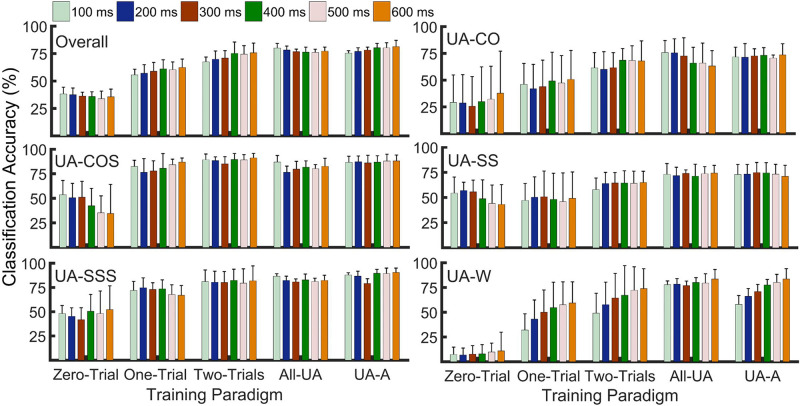
Effects of varying analysis window lengths and training paradigms on recognition accuracy of unanticipated straight walking (UA-W), cuts (UA-SS, UA-CO), and cut to stair ascent (UA-COS, UA-SSS). Zero-Trial provided the lowest accuracy in all locomotor tasks. Including a few bouts of target task significantly improved the accuracy. However, there was no statistically significant difference between Two-Trials, All-UA, and UA-A training paradigms (α = 0.01). Altering analysis window size did not result in a significant change in task recognition accuracy.

Classification of unanticipated locomotor tasks was attempted using previously introduced training paradigms and varied overlapping window lengths. Two-way ANOVA did not report any window size by training paradigm interaction effect. Window size main effect was shown to be significant only in UA-W, where increasing window length from 100 to 500/600 ms led to a slight increase (∼15%) in accuracy only in One-Trial and Two-Trail paradigms (Figure 4). No statistically significant differences in the accuracy rates of cuts/cut-to-stair transitions were observed when window length was varied within each training paradigm (*p* = 0.09–0.9). Similar results were obtained for overall accuracy across the locomotion modes.

**FIGURE 5:**
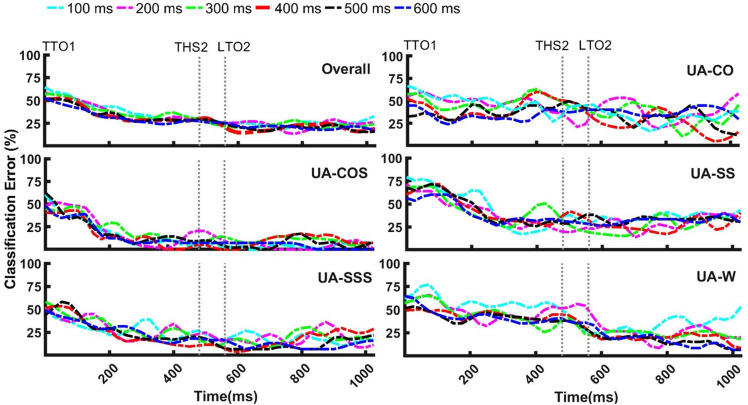
The influence of varying analysis window size on recognition of unanticipated straight walking (UA-W), cuts (UA-CO, UA-SS), and cuts to stair ascent (UA-COS, UA-SSS) over time. Highly overlapping patterns were observed for different window sizes within each locomotion mode. To execute cut, subjects started the transition at the first trailing leg toe off (TTO1) and ended with the second trailing leg heel strike (THS2). LTO2 stands for the second leading leg toe off. Events are the average across subjects. *X*-axis data was shown up to the length of the shortest trial.

To compare the impact of varied analysis window lengths over time, Two-Trials training paradigm was selected and classification error rates versus time were calculated for windows of size 100–600 ms (Figure 5). Varying window sizes demonstrated highly overlapping patterns in each locomotion mode. No significant differences other than relatively smoother patterns for the larger windows were observed. Error rates appeared to gradually decrease starting TTO1 in all locomotor tasks except in UA-CO, where high fluctuations were observed over time (Figure 5). Decreasing trends imply more accurate recognition as the tasks progress toward the subsequent events.

Training paradigm main effect was reported to be statistically significant in all locomotion modes. As indicated by post-hoc comparisons, a substantial increase (∼23%–45%) in overall accuracy was observed when training paradigms containing bouts of target task were substituted for Zero-Trial (*p* < 0.001) (Figure 4). Whereas Two-Trials, All-UA, and UA-A showed similar performances (*p* = 0.06–0.95), they outperformed One-Trial by 13%–20%. In UA-CO, including one trial of the target task did not remarkably improve the accuracy relative to Zero-Trial. Comparing the performances of training paradigms with ≥2 repetitions of target task to one another did not demonstrate any significant differences in this mode. However, they outperformed Zero-Trial by ∼18%–41%. In UA-COS, including as few as one trial of target task led to improved accuracy rates (∼36%) (Figure 4). Nonetheless, the outcomes for training paradigms containing ≥1 bout(s) of target locomotion appeared to be statistically similar. In UA-SS, One-Trial provided similar accuracy to Zero-Trial (*p* = 0.99) (Figure 4). Even though accuracy rates did not differ across training setups with ≥2 target task repetitions, they outperformed Zero-Trial by ∼13%–23%. In UA-SSS, all training paradigms with ≥1 repetition(s) of target locomotion outperformed Zero-Trial by ∼23%–40% (*p* < 0.001), whereas those with ≥2 exposures showed comparable performances. Similar results were obtained for UA-W where including ≥1 exposures of target task increased the accuracy by ∼40%–71% relative to Zero-Trial, while adding more than two bouts did not benefit the outcome (*p* = 0.2–0.5).

**FIGURE 6:**
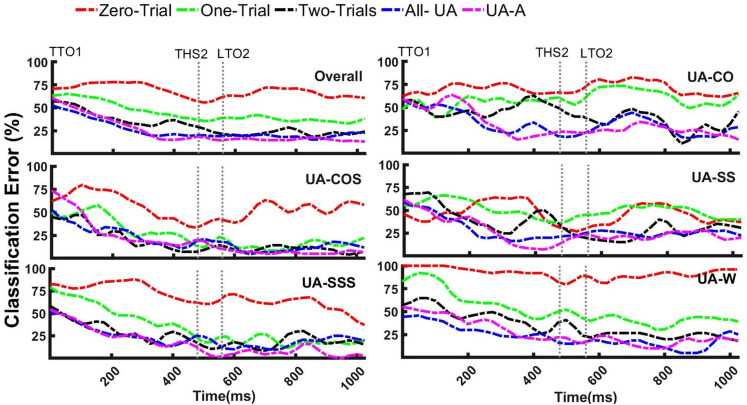
The influence of training paradigms with varying exposures of target task on recognition of unanticipated straight walking (UA-W), cuts (UA-CO, UA-SS), and cuts to stair ascent (UA-COS, UA-SSS) over time. Even though including a few bouts of target locomotion significantly decreased error rates in mixed transitions (UA-COS, UA-SSS), maximum exposures of unanticipated information were required to provide robust recognition in single transitions (UA-CO, UA-SS) and UA-W. To execute cut, subjects started the transition at the first trailing leg toe off (TTO1) and ended with the second trailing leg heel strike (THS2). LTO2 stands for the second leading leg toe off. Events are the average across subjects. *X*-axis data was shown up to the length of the shortest trial.

Within each transition, the performances of different training paradigms over time were also tested using analysis windows of nominal size 300 ms. In all locomotion modes, Zero-Trial resulted in the highest error rates over time (Figure 6). Although One-Trial and Zero-Trial paradigms showed relatively overlapping patterns in UA-CO and UA-SS, significant differences between the two patterns were observed in other modes.

Error trends for training paradigms containing ≥2 target task bouts appeared to highly overlap especially in mixed transitions (UA-COS, UA-SSS) (Figure 6). However, in single transitions (UA-CO, UA-SS) and UA-W, smoother patterns were observed for All-UA and UA-A relative to Two-Trials. In mixed transitions, error patterns for Two-Trials, All-UA, and UA-A became relatively steady around 200 ms prior to THS2 and remained mostly below 25% afterwards (Figure 6). In contrast, in single transitions, decreasing error trends were not observed, unless when the training paradigms with the maximum amount of target information (i.e., All-UA, UA-A) were used. These results suggest that only up to two bouts of target task were required to sufficiently reduce classification error in mixed transitions. According to the obtained accuracy rates for different training paradigms in single transitions/straight walking, even though two repetitions of target task appeared to be sufficient, including more target trials may be necessary to accommodate variability in classification outcome over time.

### Confusion Matrices

In order to quantify misclassification error, confusion matrices were calculated for each training paradigm using sliding and overlapping windows of nominal length 300 and 25 ms increment. In Zero-Trial, the lowest and highest accuracy levels belonged to UA-W and UA-SS, respectively (7.73% vs 55.73%) (Table 2). Using this training paradigm, UA-W was highly confused with all other transitions and most significantly with UA-SS and UA-CO by ∼35% and 30%, respectively. UA-CO and UA-SS were mistakenly recognized as each other (∼ 59%, 34%), and ∼40% misclassification was observed between UA-COS and UA-SSS. In One-Trial, UA-SS and UA-CO were largely misclassified, whereas misclassification of UA-COS and UA-SSS as each other significantly dropped to ∼12% and their recognition accuracies improved to ≥70%. Including ≥2 bouts of a given target transition was shown to remarkably reduce misclassification rates especially for UA-CO, UA-SS, and UA-W. Darker diagonal and lighter off-diagonal shades using paradigms with more target task trials are indicative of the same fact.

**TABLE 2:** Confusion matrices for unanticipated locomotor tasks using varying training paradigms.

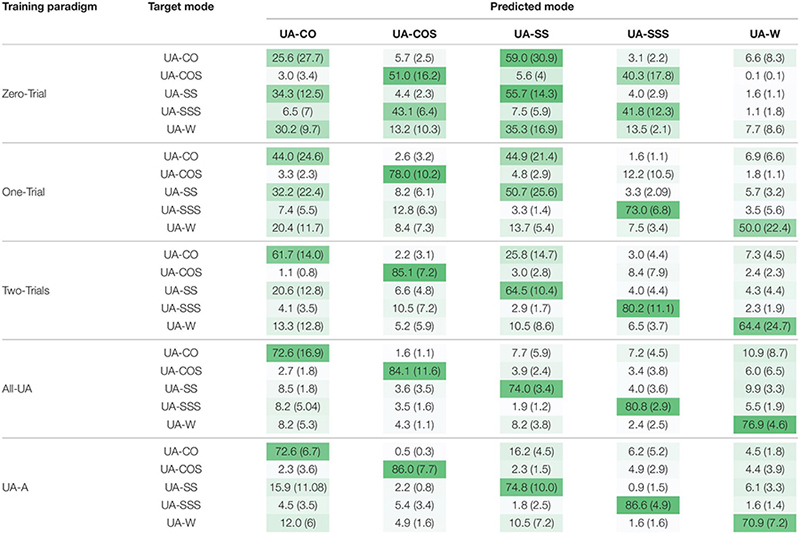

*Values represent mean (standard deviation). Diagonal elements with darker shades appeared as training paradigms with more repetitions of target task were used, with off-diagonal elements appearing lighter indicating lower misclassification level.*

## Discussion

Little is known about the influence of task anticipation and changes of direction on locomotion recognition. Increased speed during changes of direction relative to normal gait (Rand and Ohtsuki, 2000; Besier et al., 2003; Inaba et al., 2013) has been shown to decrease gait stability and cause the tasks to require increased levels of coordination especially when are unanticipated (Kang and Dingwell, 2008). These findings further emphasize the need to better inform assistive device for use during locomotor transitions, so that device utility and user safety can be improved. We introduced a set of classification schemes and tested their performances in identifying level-ground walking, 45° changes of direction (cuts), and cut to stair ascent performed under anticipated and unanticipated conditions.

Our first hypothesis that anticipatory state of the locomotor transition would impact locomotion recognition accuracy was supported. Investigating the performance of Zero-Trial approach in classifying anticipated versus unanticipated maneuvers indicated substantially different outcomes (Table 1 vs Table 2) which could be the result of substantial differences in lower extremity kinematics between the two conditions (Besier et al., 2001; Weinhandl et al., 2013; Meinerz et al., 2015). Biomechanics adaptation to the upcoming anticipatory locomotor task begins as early as three strides before the transition (Wu et al., 2015; Peng et al., 2016). Low error values prior to initiating locomotor transition (i.e., TTO1) in anticipated tasks (Figure 3), could be indicative of the modification of subject’s kinematics in the most optimal way during the preparatory phase of such maneuvers. In real-time implementation, early detection would also mean more time for an assistive device to react and support the maneuver. In contrast, unanticipated locomotor tasks were shown to be highly misclassified prior to and immediately after entering the locomotor transition (TTO1) (Figure 6). Neuromechanical adjustments of lower extremity are highly dependent on the task anticipatory state and the preparatory period preceding the transition (Meinerz et al., 2015; Wu et al., 2015). Lack of preparatory time in unanticipated maneuvers could cause high levels of kinematic dissimilarity between trials of a given transition performed under two different anticipatory states, as well as high gait variability between the trials of a particular unanticipated task (Stokes et al., 2017), resulting in poor generalization of the classification model to the test data. These could be the underlying cause of diminished accuracy rates in unanticipated locomotor tasks and imply the fact that a classification scheme designed for a given anticipatory state may not perform as well under a different state, unless the training paradigm is adapted.

We also hypothesized that including bouts of target task in the training data would lead to improved recognition of unanticipated locomotion modes. This hypothesis was supported as well. However, the level of impact was moderately different across varying locomotion modes. For instance, a given training paradigm with exposures of unanticipated target task, provided higher accuracy rates in mixed transitions (i.e., cuts to stair ascent) compared to single transitions (i.e., cuts on level ground) and straight walking locomotion (Table 2). Only two bouts of target tasks were sufficient to normalize the data to the target-task anticipatory state and provide ≥80% accuracy in recognition of mixed transitions. However, in single transitions and straight walking, even the paradigms with the maximum amount of unanticipated information appeared to have lower accuracies (∼70%–74%). In mixed transitions, using training paradigms with a few unanticipated bouts led to error trends with a significant drop prior to THS2 (Figure 6). In contrast, substantial unanticipated information was required (five bouts) to provide similar trends and relatively robust recognition of single transitions over time (Figure 6). One explanation for more accurate recognition of mixed transitions might come from significant kinematic alterations in both horizontal and vertical planes during changes of terrain (e.g., level ground to stair ascent) (Andersson et al., 1980; Rowe et al., 2000) relative to the tasks performed on level ground (e.g., single transitions). To prepare for the increased demands of terrain change, biomechanical alterations are made as early as when an individual knows they are going to make a transition (i.e., TTO1 in unanticipated tasks) (Sheehan and Gottschall, 2011; Peng et al., 2016). This results in unique differences during the transition that precedes stairs versus level ground. Increased lower-limb joints ranges of motion during mixed transitions could provide more discriminating and predictive information for the classifier, allowing relatively good generalization to unanticipated state even with minimal number of target-task bouts.

In the presence of unanticipated perturbations, kinematic variability becomes substantially higher (Stokes et al., 2017) posing a difficult classification problem as unanticipated locomotion modes have high intra-class variation (Theodoridis and Koutroumbas, 2008). However, as the number of strides following the perturbation increases, the impact of perturbation on recruited muscle synergies subsides (Dingwell et al., 2001; Chvatal and Ting, 2013). Thus, lower gait variation and higher stability are expected as the tasks progress toward the end, which could explain lower errors and more steady and robust error patterns as the transitions continue (Figure 6). The error patterns reached their minimum at approximately 200 ms prior to transitioning (trailing) leg heel strike (THS2), and remained almost steady afterwards (Figure 6), suggesting that a device worn on the trailing leg could have sufficient time to correct its mode and adjust its parameters in a smooth manner to accommodate the upcoming heel strike during unanticipated cuts, cut-to-stair ascent, and straight walking.

Our third hypothesis regarding improved accuracy with larger analysis windows was partially supported. Altering analysis windows did not result in a significant change in recognition accuracy except in UA-W, where only increasing window length from 100 to 500/600 ms led to improved outcomes. This could be due to the fact that larger window lengths contain more gait information which represent them as a more suitable option for slower locomotor tasks such as level-ground walking (Shirota et al., 2014). Highly overlapping error patterns were observed across the range of window lengths tested (Figure 5). Nonetheless, larger windows length would increase time delays in recognizing the locomotion mode, which should be taken into account in assistive device applications, where such delays could compromise the real-time control of the device.

Continuous classification utilized in this study is another factor that could explain the outcomes. The majority of control systems for lower-limb assistive devices are phase/event dependent, meaning the locomotion is identified when specific gait events occur (e.g., heel strike and toe off) (Young et al., 2014b). Since such systems cannot continuously predict user’s intended movements and there is a delay between consecutive decisions, smooth transitions between locomotor activities is challenged. Another drawback of phase-dependent approaches is their inability to handle unexpected events such as tripping (Shirota et al., 2014), or within-cycle changes of locomotor mode. In contrast, continuous classification can provide higher levels of maneuverability for the assistive device by allowing the mechanical response of the devices to be optimally adjusted during locomotion and adapt to changes according to the classifier prediction. However, since the variability of kinematic information is significantly higher across the entire trial compared to between only specific gait events, continuous classification is a more complicated case for the recognition system than event dependent. This makes it more challenging for the classifier to associate patterns in the target ambulation mode with the training data which could result in lower accuracy rates. Nonetheless, incorporating classification models allowing recursive update of classification parameters as the subject performs the task and according to new input data, could provide the system with the ability to adapt to environment changes, and could lead to improved system robustness across multiple sessions.

Overall, although correctly identifying unanticipated maneuvers is essential to prevent the users from potential stumbles and falls, handling unanticipated maneuvers is still a challenge in assistive device technology. According to the findings, including repetitions of target locomotion mode to the training data helps to normalize the classifier to the anticipatory state of the task, and was highly beneficial for reducing recognition errors of unanticipated locomotion. Whereas, altering analysis window size did not have significant influence. This study suggest that one practical approach to handle recognition of unanticipated tasks, rather than increasing classifier’s complexity, may be to adjust the training scheme where subjects execute small numbers of trials of the given task and/or to temporally adapt the classifier online based on the data that arrive continually (Spanias et al., 2017).

The study has some limitations. Only able-bodied individuals participated in this study. Future research should investigate subjects with gait impairments (e.g., lower-limb amputees) walking on assistive devices to test the generalizability of the finding of this study to individuals with mobility disorders. Human anthropometrics and their effect on human kinematics are other factors that need to be taken into consideration in future research. Studies have reported that anthropometric factors such as trunk and thigh length significantly affect knee and hip joint angles and range of motion in the lower limbs during certain tasks (Demers et al., 2018). Moissenet et al. (2019) also highlighted the fact that anthropometric parameters significantly influence gait kinematics. In our future work, we expect to investigate these effects using a larger subject pool with higher anthropometric variability.

In addition, classifier’s input data include fusion of kinematic signals from lower-limb and torso. It is possible that a subset this information would provide comparable outcomes (Kazemimoghadam and Fey, 2020) improving efficacy of the system. Future studies should attempt to reduce input signals redundancy by identifying the sources necessary for accurately recognizing the user’s intended tasks.

Biomechanical data used in this paper were extracted using a camera-based motion capture system, and we have not tested these approaches using physical sensors. Previous work has demonstrated comparable kinematics measurements using body-worn sensors and motion-capture data (Mayagoitia et al., 2002; Aziz et al., 2014), and many techniques exist to take inherent characteristics of physical sensors into consideration (Metni et al., 2006; Gallego et al., 2010; Spielvogel and Whitcomb, 2019). However, errors in actual physical sensors (e.g., inertial measurement unites, IMUs) data such as bias and drift may still affect system performance and should be considered in future studies.

## Data Availability Statement

The raw data supporting the conclusions of this article will be made available by the authors, without undue reservation.

## Ethics Statement

The studies involving human participants were reviewed and approved by Institutional Review Board. The patients/participants provided their written informed consent to participate in this study.

## Author Contributions

NF and MK performed conceptualization, methodology, software, validation, and investigation. MK performed formal analysis and data writing—original draft preparation. NF performed resources, writing—review and editing, supervision, and funding acquisition. Both authors contributed to the article and approved the submitted version.

## Conflict of Interest

The authors declare that the research was conducted in the absence of any commercial or financial relationships that could be construed as a potential conflict of interest.
