# Supplementary material for: Continuous Classification of Locomotion in Response to Task Complexity and Anticipatory State
Source: Front Bioeng Biotechnol. 2021 Apr 22;9:628050. doi: 10.3389/fbioe.2021.628050 (PMC8100249; doi:10.3389/fbioe.2021.628050)
Supplement: Supplementary file 1 [file Presentation_1.PPTX]

## Slide 1
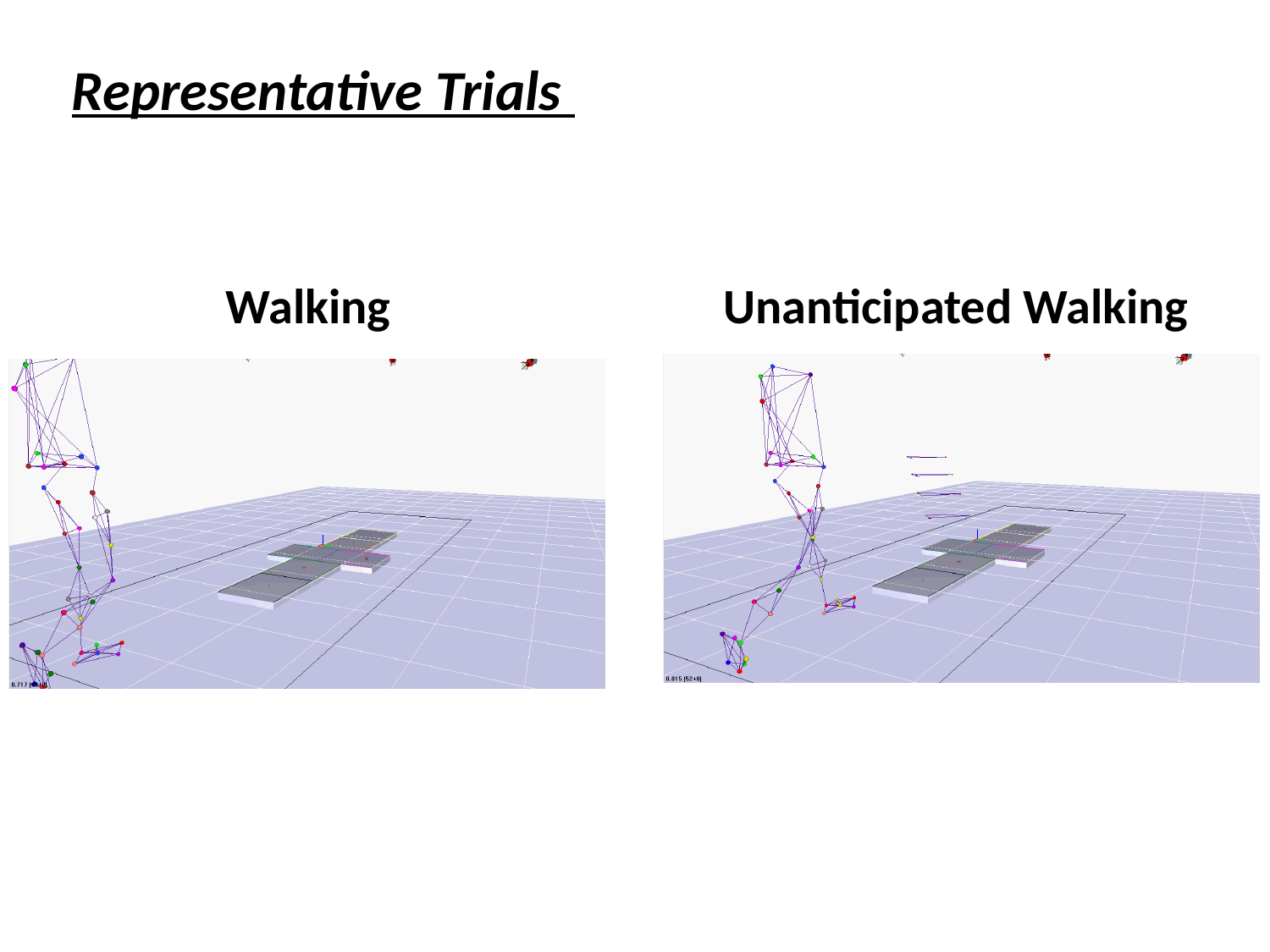

Representative Trials
Unanticipated Walking
Walking

## Slide 2
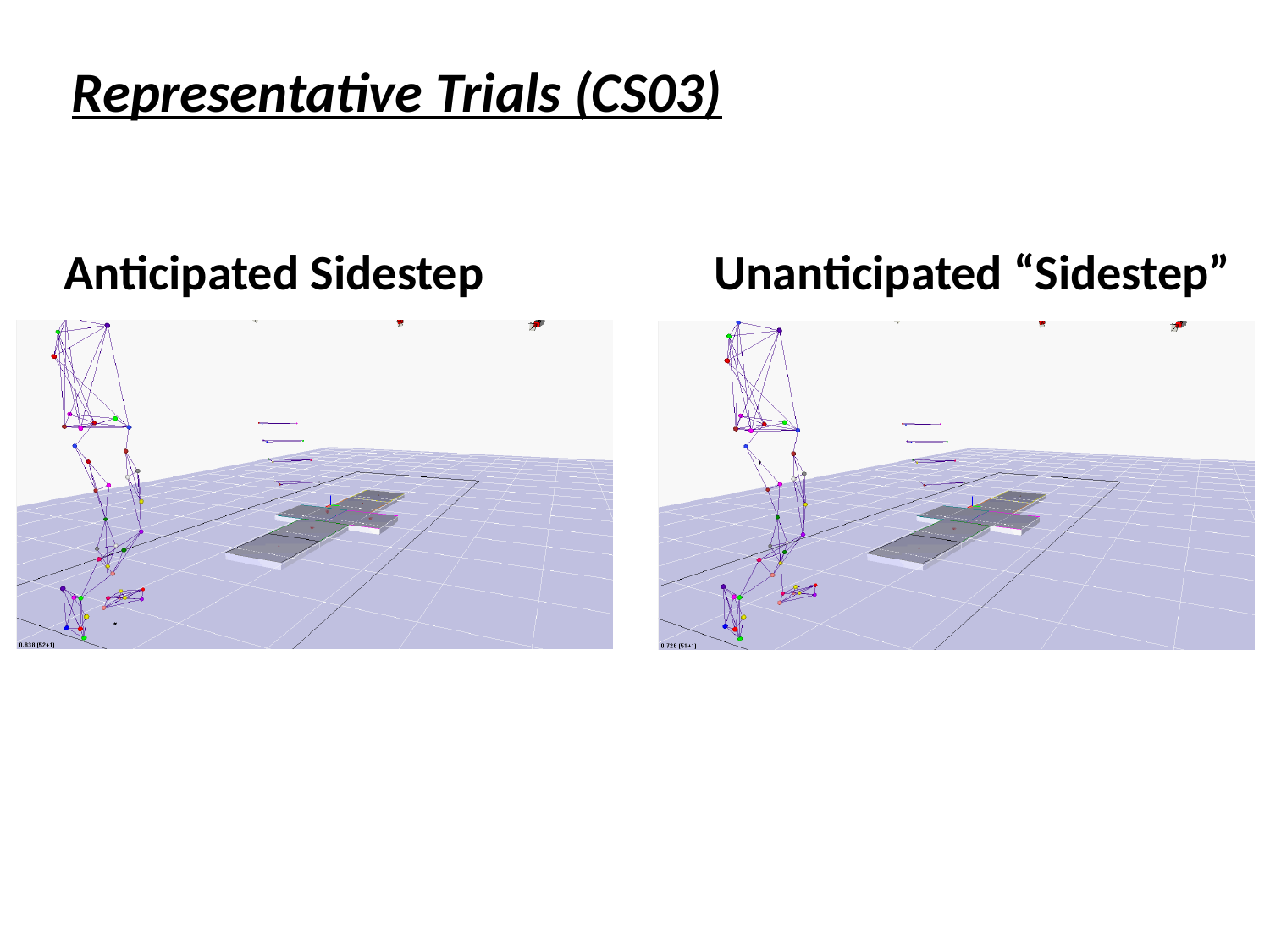

Representative Trials (CS03)
Anticipated Sidestep
Unanticipated “Sidestep”

## Slide 3
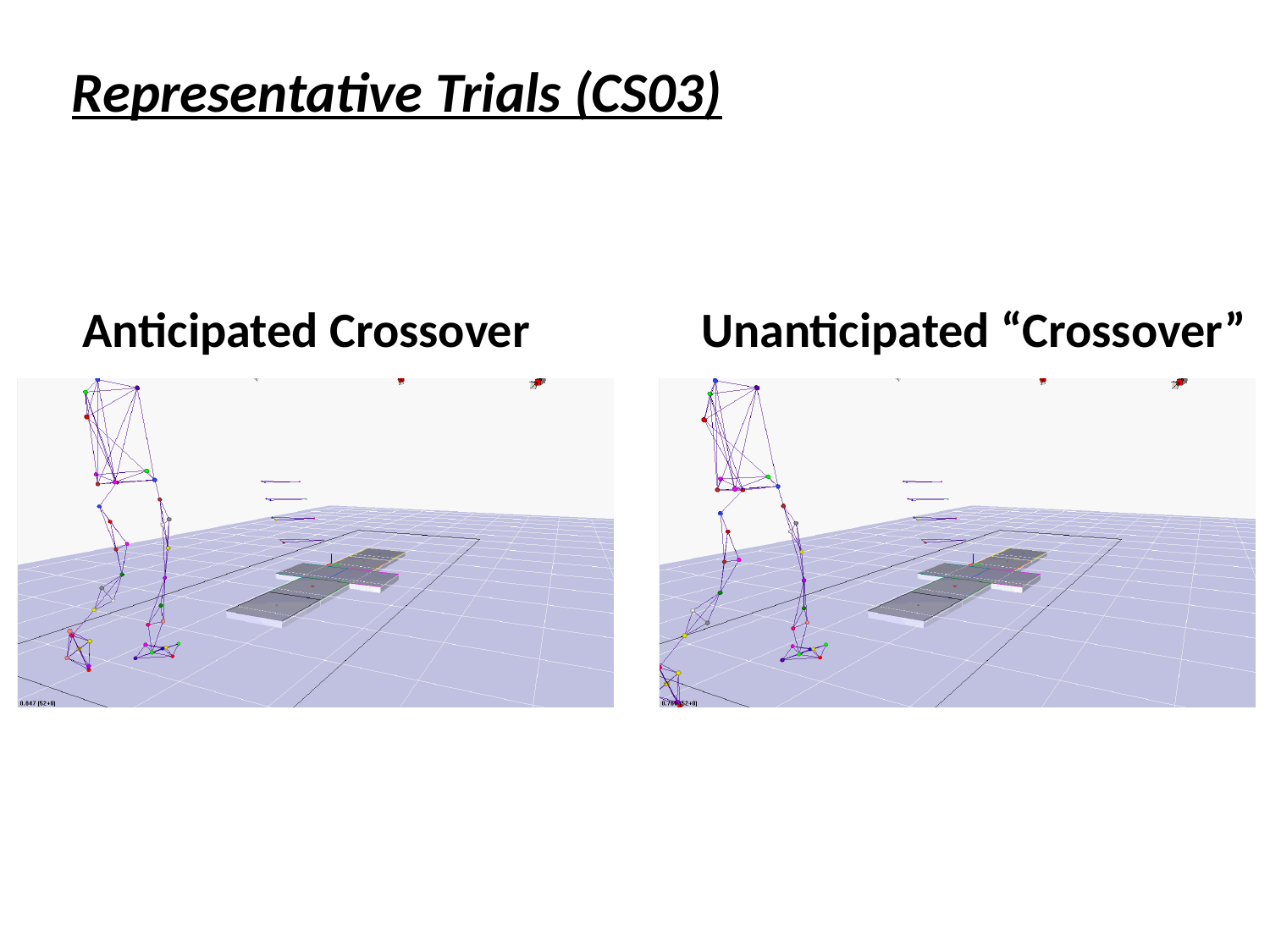

Representative Trials (CS03)
Anticipated Crossover
Unanticipated “Crossover”

## Slide 4
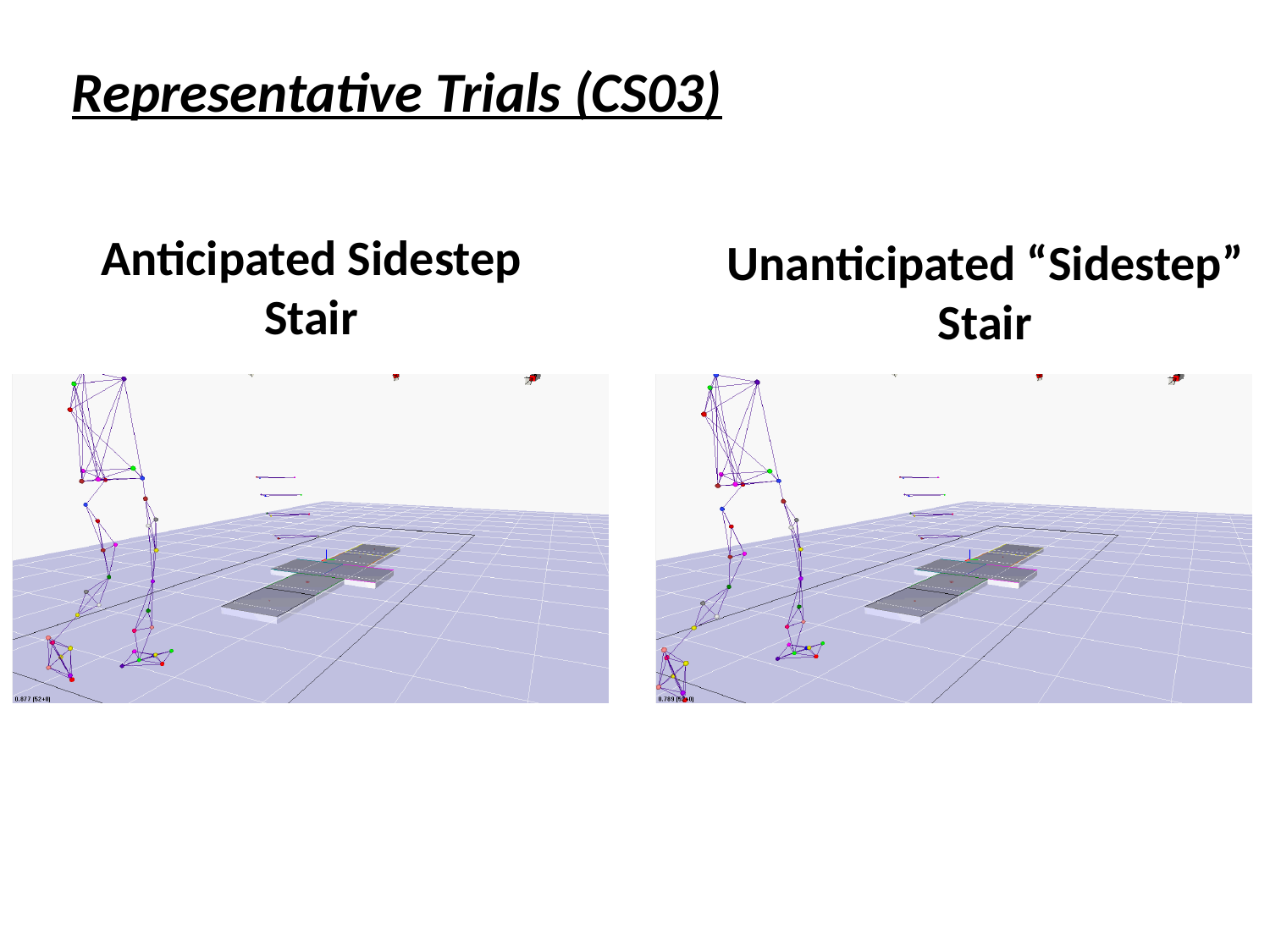

Representative Trials (CS03)
Anticipated Sidestep Stair
Unanticipated “Sidestep”
Stair

## Slide 5
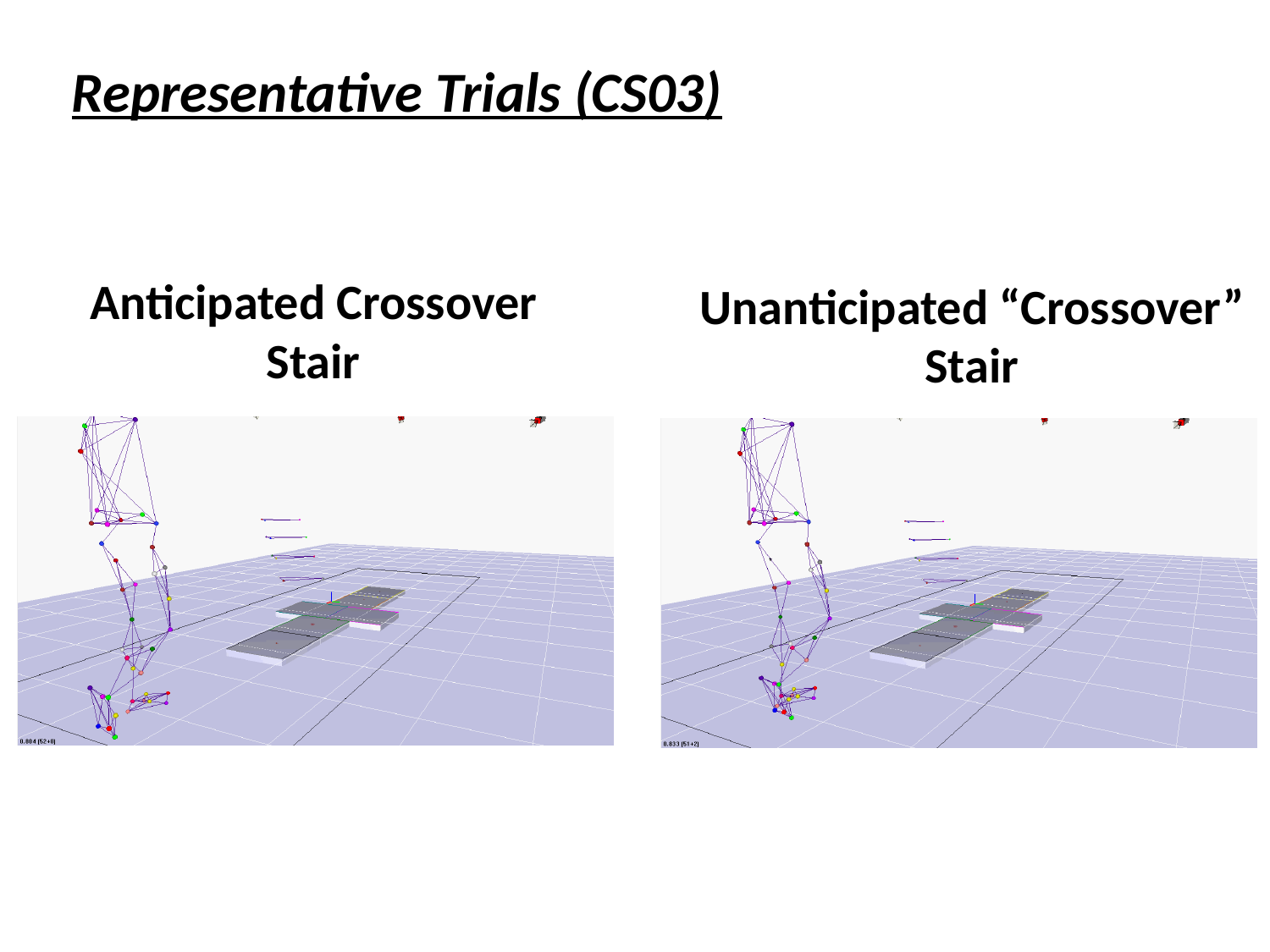

Representative Trials (CS03)
Anticipated Crossover Stair
Unanticipated “Crossover”
Stair
